# A novel immunotherapy targeting MMP-14 limits hypoxia, immune suppression and metastasis in triple-negative breast cancer models

**DOI:** 10.18632/oncotarget.17702

**Published:** 2017-05-09

**Authors:** Binbing Ling, Kathleen Watt, Sunandan Banerjee, Daniel Newsted, Peter Truesdell, Jarrett Adams, Sachdev S. Sidhu, Andrew W.B. Craig

**Affiliations:** ^1^ Department of Biomedical and Molecular Sciences, Queen's University, Cancer Biology and Genetics Division, Queen's Cancer Research Institute, Kingston, ON, Canada; ^2^ The Donnelly Centre, University of Toronto, Toronto, ON, Canada

**Keywords:** MMP-14, inhibitory antibody, breast cancer, hypoxia, metastasis

## Abstract

Matrix metalloproteinase-14 (MMP-14) is a clinically relevant target in metastatic cancers due to its role in tumor progression and metastasis. Since active MMP-14 is localized on the cell surface, it is amenable to antibody-mediated blockade in cancer, and here we describe our efforts to develop novel inhibitory anti-MMP-14 antibodies. A phage-displayed synthetic humanized Fab library was screened against the extracellular domain of MMP-14 and a panel of MMP14-specific Fabs were identified. A lead antibody that inhibits the catalytic domain of MMP-14 (Fab 3369) was identified and treatment of MDA-MB-231 breast cancer cells with Fab 3369 led to significant loss of extracellular matrix degradation and cell invasion abilities. In mammary orthotopic tumor xenograft assays, MMP-14 blockade by IgG 3369 limited tumor growth and metastasis. Analysis of tumor tissue sections revealed that MMP-14 blockade limited tumor neoangiogenesis and hypoxia. Similar effects of MMP-14 blockade in syngeneic 4T1 mammary tumors were observed, along with increased detection of cytotoxic immune cell markers. In conclusion, we show that immunotherapies targeting MMP-14 can limit immune suppression, tumor progression, and metastasis in triple-negative breast cancer.

## INTRODUCTION

Breast cancer afflicts one in eight women in North America, and is a leading cause of cancer-related death in women [1]. Triple-negative breast cancer (TNBC) represents 10–20% of breast cancer cases, and lack expression of estrogen receptor (ER), progesterone receptor (PR) and human epidermal growth factor receptor 2 (HER2) [2]. TNBCs have high rates of metastasis [3], and lack targeted therapies such as those for luminal and HER2 subtypes [4, 5]. Currently, TNBC patients undergo combination therapies consisting of surgery, chemotherapy and radiotherapy, but have worse survival rates compared to patients with other subtypes [6]. To improve outcomes for TNBC, it will be important to identify clinically relevant targets and develop new targeted therapies.

Metastasis is the leading cause of cancer-related deaths [7], and this process involves cancer cells gaining the ability to degrade basement membranes and spread to other tissues via blood or lymphatic vessels [8]. Targeting early steps in metastasis, such as extracellular matrix (ECM) degradation and invasion in cancer cells, could improve outcomes in TNBC [9]. The matrix metalloproteinase (MMP) family mediate ECM degradation and promote cancer metastasis [10, 11]. Over the last two decades, several broad spectrum, small molecule MMP inhibitors have been developed and tested in cancer clinical trials. However, these inhibitors have failed due to adverse side effects due to lack of selectivity for these small molecules targeting the catalytic domains of MMPs that are highly conserved [12]. To overcome this, it may be possible to develop highly selective inhibitory antibodies to individual MMPs that are active on the surface of metastatic tumors, or within the tumor microenvironment (TME). MMPs are localized to invadopodia, which are filamentous actin (F-actin)-rich cellular protrusions that degrade ECM [13]. Invadopodia formation requires MMP-14, a cell surface receptor that degrades collagen, and activates secreted MMPs, to promote cancer metastasis [14]. As expected, increased expression of MMP-14 leads to increased metastasis in cancer models, and also correlates with poor prognosis in human breast cancer patients [15].

The localization of MMP-14 is highly regulated, with storage in secretory endosomes that can be trafficked to the plasma membrane at sites of invadopodia formation [16]. High expression of MMP-14 on the surface of cancer cells is facilitated by Src and FAK kinases that suppress the endocytic machinery [17]. Fast recycling of MMP-14 also promotes invadopodia formation in cancer cells [16]. The high levels of MMP-14 on the surface of metastatic cancer cells make it an excellent target for cancer therapy development [18]. The extracellular domain (ECD) of MMP-14 includes both a hemopexin (Hpx) domain, and a zinc-dependent protease domain, and both domains are required for function and potential sites of targeting by inhibitors [19, 20].

Here, we screened a synthetic antibody library to identify inhibitory MMP-14 antibodies. Our lead inhibitory clone 3369, when used as an antigen-binding fragment (Fab) or as an immunoglobulin (IgG), suppressed MMP-14 activity *in vitro* and displayed low nM affinity. Treatment of TNBC cells with Fab impaired ECM degradation and cell invasion. In mammary tumor models, treatment with IgG 3369 led to suppression of hypoxia and a skewed TME that limited tumor progression and metastasis.

## RESULTS

### Selection of synthetic MMP-14 antibodies

**Figure 1:**
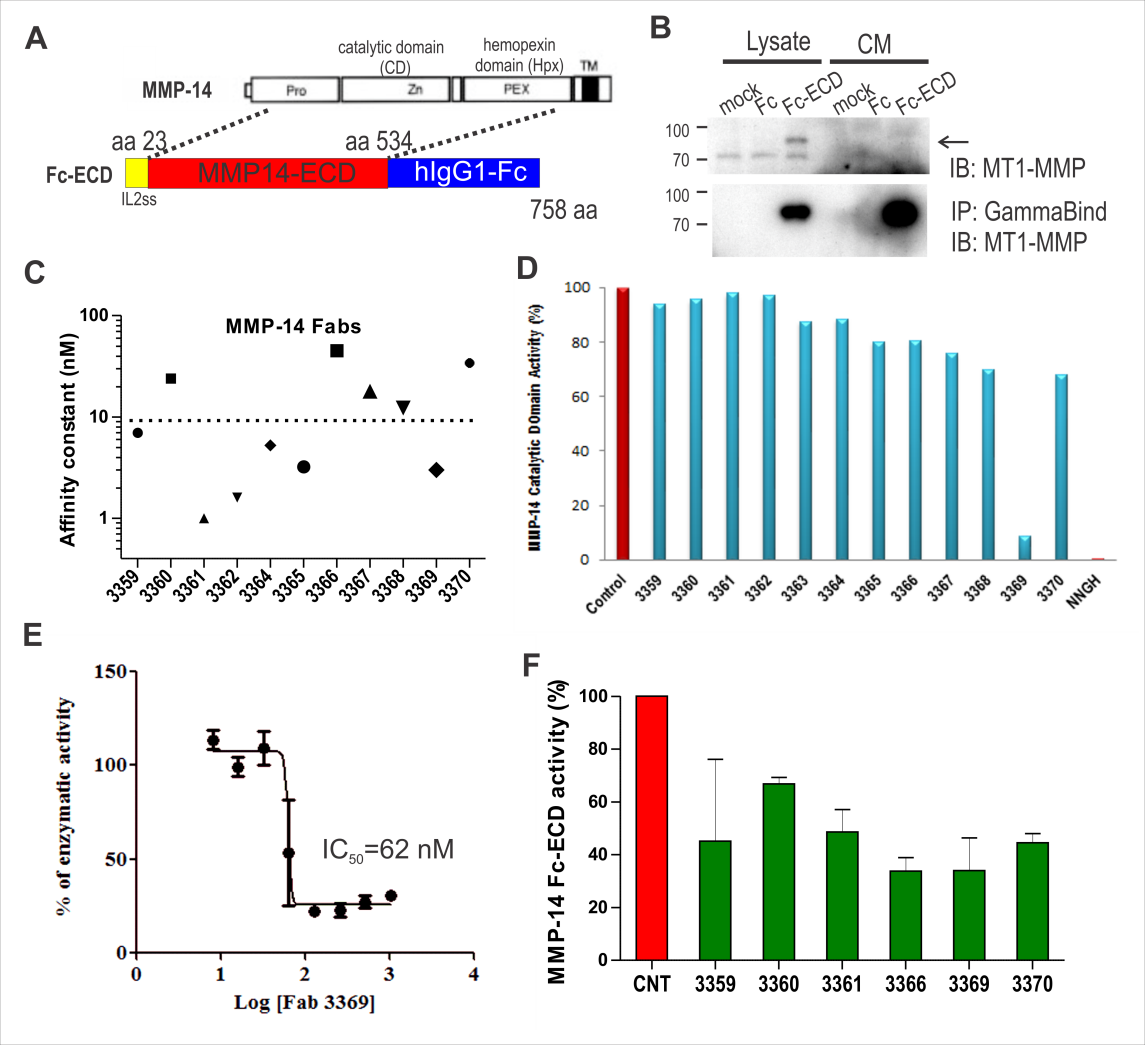
Anti-MMP-14 synthetic antibody identification and profiling (**A**) Schematic of MMP-14 extracellular domain (ECD) fusion with Fc and IL-2 signal sequence (ss) encoding amino acids (aa) 23-534 of human MMP-14 spanning the prodomain, catalytic domain (CD) and hemopexin (Hpx) domain (but not the transmembrane (TM) domain). (**B**) Test expression of Fc-ECD in HEK293T cells using lysates and conditioned media (CM). MMP-14 ECD-Fc fusion protein was captured using GammaBind sepharose and detected by IB with anti-MMP14 antibody. (**C**) The affinity constants (K_D_) for purified anti-MMP-14 Fabs binding to the MMP-14 ECD-Fc construct were determined by surface plasmon resonance. (**D**) Inhibition of MMP-14 protease activity (initial rate) was measured *in vitro* using a commercial quenched fluorogenic MMP substrate incubated with MMP-14 catalytic domain with or without the indicated anti-MMP14 Fabs (800 nM). The small molecule inhibitor NNGH (1.3 μM) was used as a control. (**E**) The dose dependence of Fab 3369 on MMP-14 activity (initial rate) was measured as above (IC_50_ = 62 nM). (**F**) Trypsin-activated MMP-14 ECD-Fc fusion protein (185 nM) was used as the enzyme incubated with quenched fluorogenic MMP substrate in the absence or presence of the indicated anti-MMP14 Fabs (1 μM). The changes in the initial rate relative to no Fab control are shown.

To develop improved tools for the blockade of MMP-14 activity, we produced a construct expressing the extracellular domain (ECD) of MMP-14 fused to the human IgG1 Fc domain (Figure 1A). Upon transient transfection in HEK293T cells, the construct was effectively captured from conditioned media using GammaBind sepharose beads and detected by immunoblot (Figure 1B). Following large scale expression and affinity purification with Protein A sepharose beads, the Fc-ECD construct was used as bait protein for screening of a phage-displayed synthetic humanized Fab library [21]. Following 4 rounds of sequential negative and positive selection using Fc and Fc-ECD, respectively, DNA sequencing of ELISA positive clones revealed 12 unique clones. These 12 clones were subcloned for expression as free Fab proteins. We detected selective binding of all Fab's to MMP-14 Fc-ECD construct relative to Fc in an ELISA format (data not shown), and performed surface plasmon resonance assays to define the binding constants (K_D_) of the selected Fabs. These assays revealed several Fabs that had low nM affinity (Figure 1C).

To screen for inhibitors, Fab proteins were tested for effects on MMP-14 protease activity towards a quenched fluorogenic substrate. When screened against the purified catalytic domain of MMP-14, only one Fab (denoted 3369) showed a significant suppression of activity that was similar to that of a small molecule pan MMP inhibitor NNGH (Figure 1D). Further titration of Fab 3369 in the activity assays using the MMP-14 catalytic domain revealed an IC_50_ value of 62 nM (Figure 1E). We also tested the Fab's using the larger MMP-14 Fc-ECD construct, and this further validated Fab 3369 as an inhibitor, but also expanded the number of inhibitory Fab's to several other clones that may inhibit by binding to epitopes outside of the catalytic domain (Figure 1F). Together, these results identify Fab 3369 as a lead inhibitory synthetic antibody that targets the catalytic domain of MMP-14.

### MMP-14 blockade by Fab 3369 inhibits ECM degradation and TNBC cell invasion

**Figure 2:**
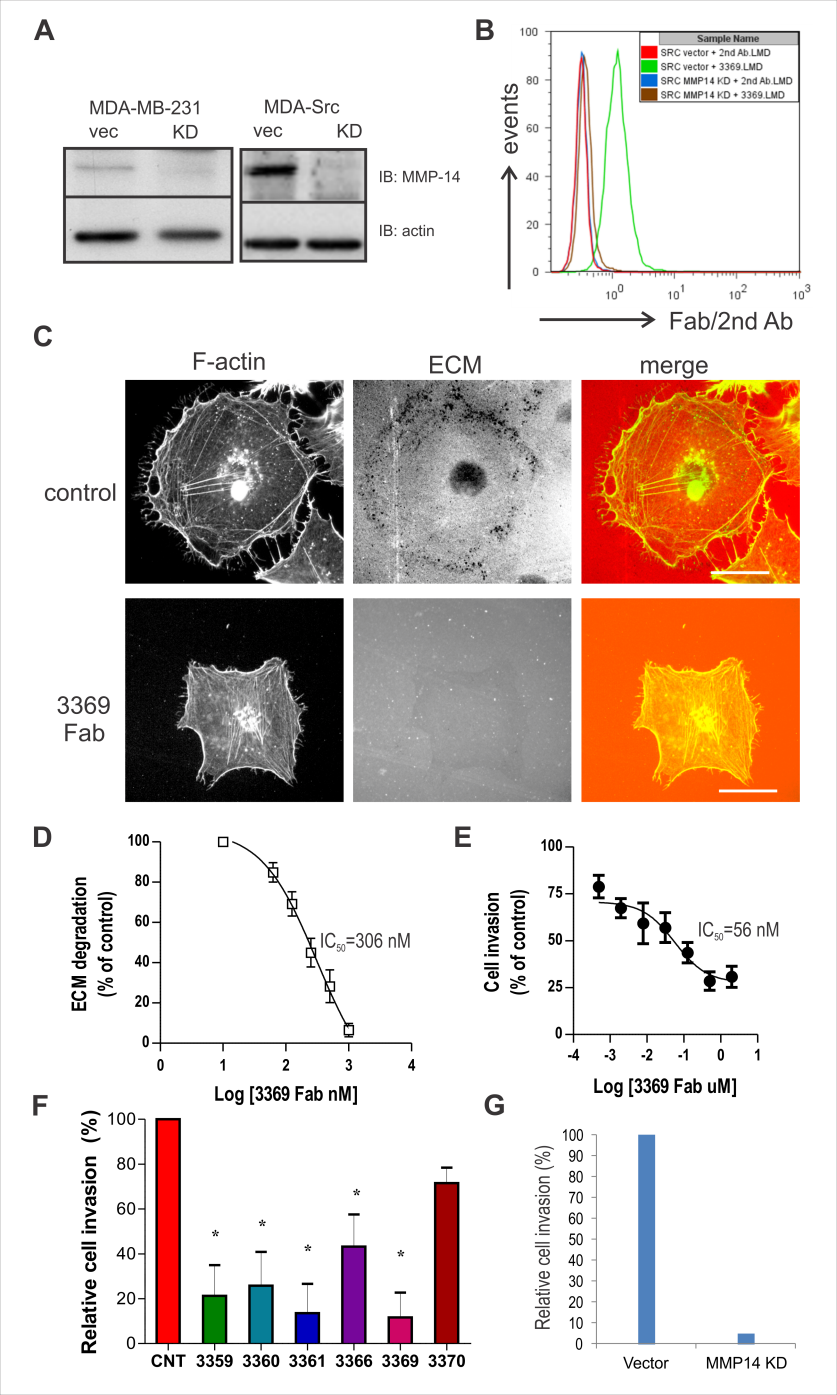
Fab 3369 inhibits MMP-14-mediated ECM degradation and MDA-MB-231 cell invasion (**A**) Immunoblots were performed to assess protein levels of MMP14 in MDA-MB-231 cells and MDA-Src cells transduced with lentiviral vector control (vec) or with a vector expressing shRNA to generate a stable MMP14 knock-down (KD; β-actin served as a loading control). (**B**) Histogram overlay of flow cytometry results for IgG 3369 binding to MMP-14 on the surface of MDA-Src vector cells or KD cells using a fluorescent secondary antibody. (**C**) MDA-Src cells were seeded on TRITC-gelatin-coated coverslips and treated with or without Fab 3369 (500 nM) for 8 hours. Cells were fixed, stained with Phalloidin (F-actin) and representative epifluorescence images are shown (scale bars: 30 μm). (**D**) Graphs depict quantification of ECM degradation by Fab 3369 relative to untreated control cells. (**E**) The effects of Fab 3369 treatment (0.5 nM-2 μM) on the invasion of MDA-Src cells through matrigel-coated transwell filters was assessed at 24 hours. Graphs depict DAPI-stained cells relative to untreated control cells (mean ± SEM of three independent experiments performed in triplicate). (**F**) The effects of treating MDA-Src cells with other inhibitory anti-MMP-14 Fabs (1 μM) were compared in cell invasion assays as described above (*indicates *p <* 0.05 compared to control (CNT) based on one way ANOVA). (**G**) The effects of MMP-14 KD on MDA-Src cell invasion is shown in parallel cell invasion assays.

To test the selectivity of our lead inhibitory Fab 3369 for endogenous MMP-14, we used lentiviral shRNA to select for stable MMP-14 knock-down (KD) in MDA-MB-231 cells, and in a derivative cell line expressing constitutively active Src (MDA-Src) [22]. Lysates were subjected to immunoblot with MMP-14 antisera, which revealed an almost complete silencing of MMP-14 in both cell lines (Figure 2A; actin served as a loading control). Using MDA-Src cells that express high levels of MMP-14 on the cell surface [22], we tested binding of Fab 3369 to MDA-Src vector control and MMP-14 KD cells. Flow cytometry analysis indicated that all vector control cells were positive for Fab 3369 binding, whereas no binding was observed in KD cells (Figure 2B). Similar results were observed in MDA-MB-231 vector and KD cells (data not shown). These results demonstrate the selectivity of Fab 3369 towards endogenous MMP-14 expressed on the cell surface of aggressive TNBC cells.

MDA-Src cells form invadopodia and degrade ECM in an MMP-14-dependent manner [23], and we used this assay to test the effects of Fab 3369 on MMP-14 activity *in situ*. MDA-Src cells were plated on thin fluorescent gelatin-coated coverslips in the presence of either a control IgG or Fab 3369 (500 nM) for 6 hours. Following fixation, permeabilization and staining of F-actin using phalloidin, epifluorescence microscopy revealed extensive ECM degradation spots near the cell periphery for control IgG (Figure 2C, upper panels). In contrast, very limited ECM degradation was visible for Fab 3369 treated MDA-Src cells (Figure 2C, lower panels). A dose response analysis of Fab 3369 treatment effects on ECM degradation revealed an IC_50_ value of ~300 nM (Figure 2D). To test if Fab 3369 treatment is capable of a sustained blockade of MMP-14 activity, we performed a dose response analysis of TNBC cell invasion using Matrigel-coated transwell filters. These experiments revealed a dose-dependent suppression of MDA-Src cell invasion at 48 hours post treatment with Fab 3369 (Figure 2E, IC_50_ = 56 nM). In addition, similar defects in MDA-Src cell invasion were observed for the other inhibitory Fab's (Figure 2F), and to a similar extent to that of MMP-14 KD (Figure 2G), It is worth noting, that MMP-14 blockade or silencing had no overt effects on cell growth or viability in TNBC cells (Supplementary Figure 1). Together, these results demonstrate that anti-MMP-14 Fab 3369 prevents MMP-14 activation required for TNBC cells to degrade ECM, and invade collagen-rich ECM barriers *in vitro*.

### MMP-14 blockade with IgG 3369 impairs human TNBC tumor growth and metastasis

**Figure 3:**
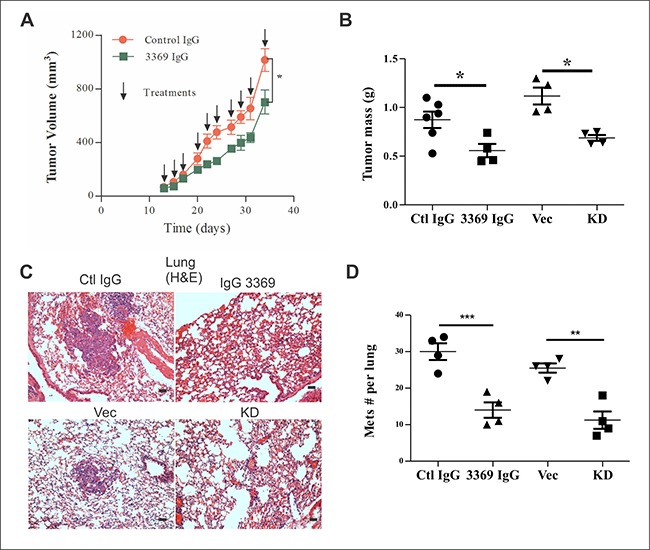
MMP-14 inhibitory antibody 3369 limits MDA-MB-231 tumor xenograft growth and metastasis (**A**) Mammary orthotopic MDA-MB-231 breast tumor xenograft assays were performed, as described in Materials and Methods, and randomized between treatments with either control IgG or IgG 3369 (5 mg/kg, intraperitoneal (i.p.) injections, 3 times/week; *n* = 6/group) following palpable tumor detection. Tumor volumes were calculated until endpoint was reached in control group (arrows indicate treatment times). (**B**) At endpoint, the tumor mass was measured for treatment groups, and compared to that of a parallel study of MDA-MB-231 vector (Vec) and MMP-14 KD tumor xenograft tumors (*n* = 4 for Vec and KD; * indicates *p* < 0.05 based on one way ANOVA comparing treatment groups and KD). (**C**) Representative hematoxylin/eosin (H&E) staining of lung tissue sections from MDA-MB-231 tumor-bearing mice comparing Vector (Vec) with MMP-14 KD, or treatments with control (Ctl) IgG or IgG 3369 (Scale bars: 50 μm). (**D**) Total numbers of lung metastases were scored from H&E-stained tissue sections. Significant differences in lung metastases were detected between treatment groups and with silencing of MMP-14 (*n* = 4 animals per group, ** indicates *p* < 0.01, or ***p < 0.001, based on one way ANOVA comparing treatment groups and KD).

To further test our lead inhibitory MMP-14 antibody, we purified this clone as a human IgG1, and tested it in mammary orthotopic xenograft assays using MDA-MB-231 cells injected in recipient mice lacking NK, B and T cells (Rag2^–/–^:IL2Rɣc^–/–^) [24]. Upon detection of palpable tumors, mice were randomized between control IgG (gammaglobulin) and IgG 3369 treatment groups (5 mg/kg; intraperitoneal injections every 2–3 days). Measurements of tumor volumes showed that IgG 3369 treatment caused a significant reduction in tumor growth rate (Figure 3A). This treatment effect was also supported at endpoint, with reduced tumor mass for the IgG 3369 treatment group (Figure 3B). Interestingly, the differences with MMP-14 blockade were similar to results from parallel studies of MDA-MB-231 vector and MMP-14 KD tumors (Figure 3B). Analysis of lung tissue sections from these mice revealed more lung metastases in the control IgG and vector control groups compared to the IgG 3369 treated or MMP-14 KD groups (Figure 3C). Scoring of the lung metastases in this model revealed significant reductions in lung metastases with MMP-14 blockade or KD (Figure 3D). Together, these results demonstrate the efficacy of MMP-14 blockade by our lead inhibitory synthetic antibody in a human TNBC xenograft model.

### MMP-14 blockade disrupts the hypoxic TNBC tumor microenvironment

**Figure 4:**
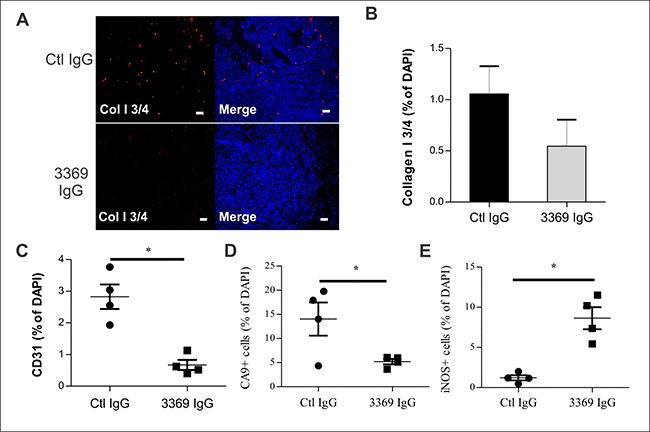
MMP-14 blockade alters the tumor microenvironment (**A**) Mammary orthotopic MDA-MB-231 tumor cryosections were prepared from control IgG or IgG 3369 treated mice, and stained with an antibody detecting MMP-mediated cleavage of Collagen I (Col I ¾; DAPI staining of nuclei was used to detect tumor area; Scale bars: 50 μm). (**B**) Graphs depict scoring of Col I ¾ staining density (relative to DAPI) between treatment groups (*n* = 4 per group). C-E) Effects of treatment with IgG 3369 on staining of CD31+ cells (**C**), CA9+ cells (**D**), and iNOS+ cells (**E**) were analyzed in tumor tissue cryosections (counterstained with DAPI). The images were acquired by epifluorescence microscopy and image analysis was performed to compare staining density (relative to DAPI) between treatment groups (*n* = 4 per group; *indicates *p* < 0.05). For representative images see Supplementary Figure 2).

To better define the effects of MMP-14 blockade with IgG 3369 on the TME, cryosections were prepared for staining with markers of MMP-14 activity and the TME. To test for effects of IgG 3369 treatment on MMP-14 activity *in vivo*, tumor cryosections were stained with cleaved Collagen I ¾ (Col I ¾) fragment-specific antibody and visualized by immunofluorescence. The relative density of cleaved Col I ¾ was higher in tumors from the control IgG group compared to the IgG 3369 group (Figure 4A, 4B). This is consistent with an effective blockade of MMP-14 activity within the TME for the IgG 3369 treatment regimen. To define the consequence of MMP-14 blockade within the TME, we examined markers of tumor vasculature (CD31), hypoxia (CA9), and a marker of anti-tumor M1 tumor-associated macrophages (TAMs; iNOS). Compared to the control IgG treatment group, mice treated with IgG 3369 had tumors with reduced density of CD31+ blood vessels and CA9+ tumor cells (Figure 4C, 4D; see Supplementary Figure 2 for representative images). Reduced hypoxia marker levels may reflect a normalizing effect of MMP-14 blockade on the properties of these blood vessels. In contrast, the density of iNOS+ cells increased in tumors form the IgG 3369 treatment group (Figure 4E; see Supplementary Figure 2 for representative images). Further profiling of gene expression changes between treatment groups showed that IgG 3369 treatment led to altered expression of genes linked to angiogenesis, TGFβ signaling, tumor growth and invasion (Supplementary Figure 3). These results are consistent with MMP-14 blockade limiting features of the TME that promote TNBC progression and metastasis.

### MMP-14 blockade impairs tumor progression and metastasis in a syngeneic breast cancer model

**Figure 5:**
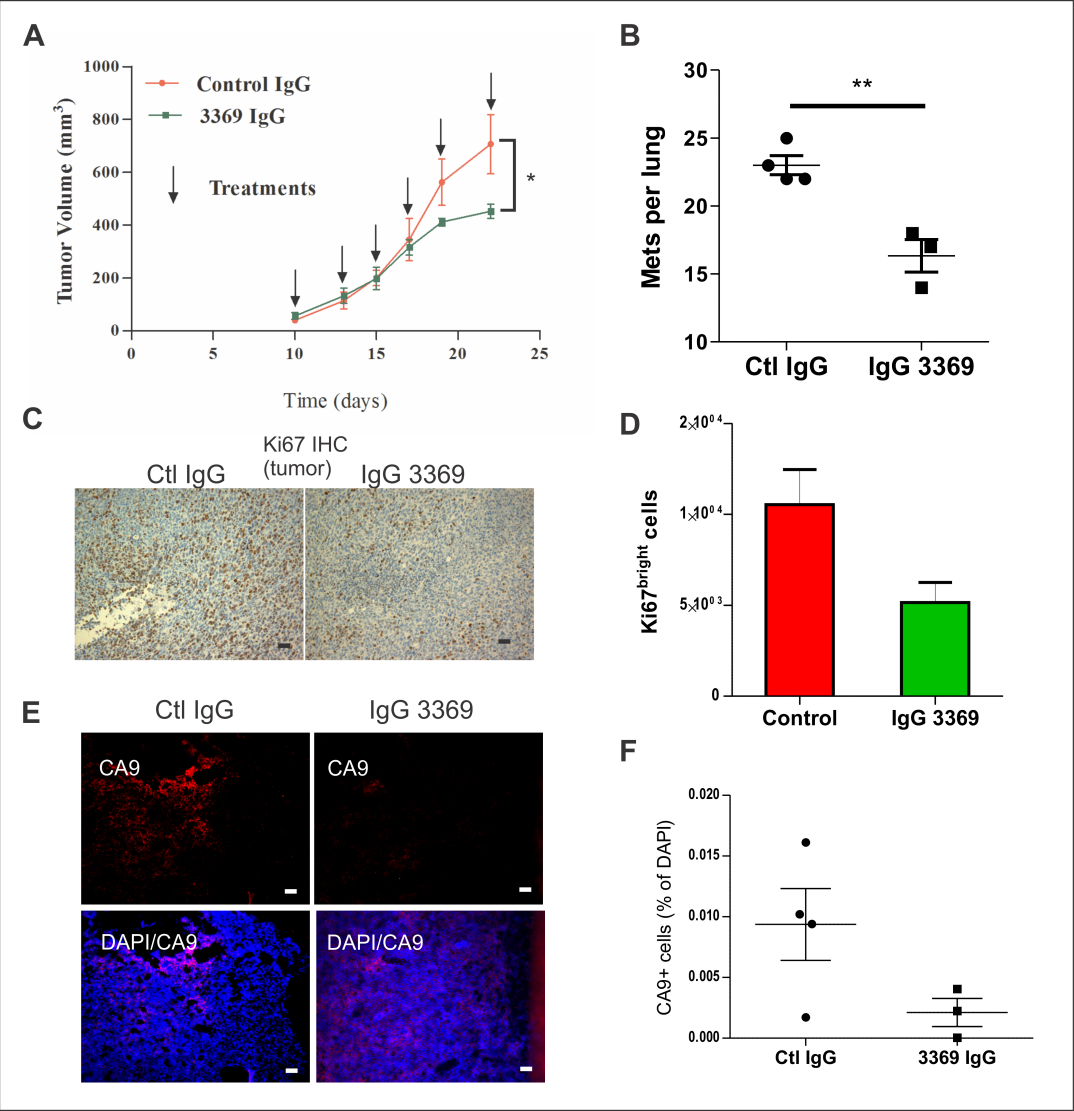
MMP-14 blockade limits tumor progression and metastasis in a syngeneic TNBC model (**A**) Syngeneic engraftment of 4T1 mouse mammary carcinoma cells in mammary glands of Balb-c mice was performed, and upon detection of palpable tumors, mice were treated with either IgG 3369 or control IgG (5 mg/kg; i.p. every 2-3 days; *n* = 6 animals per group that were derived from two independent experiments). Graph depicts tumor volumes for each treatment group until humane endpoint was reached (arrows indicate the times of treatment). (**B**) Total numbers of lung metastases from the above treatment groups were scored from lung tissue sections stained with Ki67. (**C**–**D**) The relative proliferation index of primary 4T1 tumors from each treatment group were analyzed by IHC staining with anti-Ki67. Representative images are shown (C) as well as quantification using imaging software (D). (**E**–**F**) Effects of IgG 3369 treatment on hypoxia in 4T1 tumors was assessed by staining of cryosections with CA9 antibody. Representative images of CA9 and merge with DAPI are shown (E), along with a graph showing quantification of CA9+ cells relative to DAPI-stained tumor area.

To more comprehensively profile the effects of MMP-14 blockade on anti-tumor immunity, we used the syngeneic model of 4T1 cell engraftment in the mammary fat pad of Balb-c mice [25]. When palpable tumors were observed at 10 days post injection, mice were randomized between control IgG and IgG 3369 treatment groups (5 mg/kg; intraperitoneal injections every 2–3 days). Tumor growth rates were similar between groups initially, but final tumor volume was significantly lower at endpoint for the group treated with IgG 3369 in this rapidly progressing TNBC model (Figure 5A). The numbers of lung metastases were also significantly reduced with 3369 treatment in this syngeneic breast cancer model (Figure 5B). Peripheral blood analysis revealed no evidence of toxicity with IgG 3369 treatment in these mice (Supplementary Table 1).

Staining of tumor tissue sections revealed that MMP-14 blockade reduced the percentage of tumor cells positive for the proliferation marker Ki67 (Figure 5C, 5D). Furthermore, immunofluorescent staining of tumor cryosections with anti-CA9, a hypoxia marker, revealed that MMP-14 blockade reduced hypoxia in 4T1 tumors (Figure 5E, 5F). Together, these results demonstrate that 4T1 tumor growth, hypoxia and metastasis are limited upon MMP-14 blockade.

**Figure 6:**
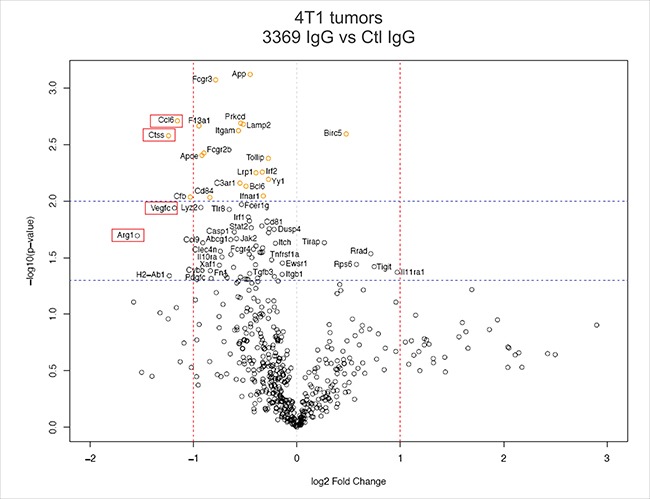
MMP-14 blockade alters the immune microenvironment of 4T1 tumors A volcano plot was generated to analyze differential gene expression in syngeneic 4T1 tumors between control IgG and IgG 3369 treatment groups (*n* = 6 per group from two independent experiments). This involved hybridization of tumor RNA to the mouse pan cancer immune codeset (nanoString) followed by defining probe counts and bioinformatics analysis, as described in Materials and Methods (red dashed lines indicate > 2-fold change, and blue lines indicate *p* < 0.05 and *p* < 0.01 levels of significance; red boxes indicate differentially expressed genes referred to in the text).

To more comprehensively profile the immune TME in these tumors, transcriptome profiling was performed using mouse pan cancer immune panel screening (nanoString). Differential gene expression analysis (*n* = 6 tumors per group) revealed a number of immune regulatory genes altered with MMP-14 blockade (Table 1, Figure 6A). Several highly downregulated genes in tumors treated with IgG 3369 were identified as the M2-TAM marker Arg1 (-2.92 fold change), granulocyte marker Itgam (-1.48 fold change), and macrophage activation marker Cd82 (-1.79 fold change). The chemokine Ccl6 (-2.23 fold change) and the angiogenesis-promoting growth factor Vegfc (-2.27 fold change) were also downregulated upon MMP-14 blockade (Table 1, Figure 6A). KEGG pathway analysis revealed phagosome, cytokine receptors, and Jak-STAT pathways as the most highly represented in the differentially expressed genes with MMP-14 blockade (Supplementary Figure 4). To test whether skewed expression of these immune markers were validated at the level of immune cell staining within the TME, tumor cryosections were stained with markers associated with TAMs and cytotoxic T cells. MMP-14 blockade led to increased iNOS+ cells and reduced CD206+ cells with the mammary tumors (Supplementary Figure 5A, 5B). In addition, the density of Granzyme B+ cells were significantly increased in tumors from the IgG 3369 treatment group compared to control IgG (Supplementary Figure 5C, 5D). Together, these results highlight the potential of MMP-14 blockade to disrupt the immune suppressive TME in metastatic breast cancers.

**Table 1:** Most significantly altered genes in 4T1 mammary tumors treated with anti-MMP-14 inhibitory antibody 3369 versus control IgG

Gene	*p* value	Fold Change	Biological Process
**App**	7.60E-04	–1.37	Cell recognition, cellular calcium ion homeostasis
**Fcgr3**	8.50E-04	–1.72	Immunoglobulin mediated immune response, leukocyte chemotaxis,
**Ccl6**	1.96E-03	–2.23	Cell chemotaxis, chemokine receptor binding, cytokine receptor binding
**Prkcd**	2.05E-03	–1.46	Adaptive immune response, neutrophil activation
**Lamp2**	2.10E-03	–1.43	Protein targeting to lysosome involved in chaperone-mediated autophagy
**F13a1**	2.17E-03	–1.93	Blood coagulation, blood microparticle, coagulation, hemostasis
**Itgam**	2.39E-03	–1.48	Granulocyte adhesion and chemotaxis, macrophage differentiation
**Birc5**	2.55E-03	1.39	Negative regulation of neuron apoptotic process
**Ctss**	2.65E-03	–2.37	Positive regulation of inflammatory response, angiogenesis, tumor progression
**Fcgr2b**	3.77E-03	–1.87	Leukocyte mediated immunity, negative regulation of leukocyte activation
**Apoe**	3.95E-03	–1.89	Regulation of cell growth, regulation of cancer stem cells, hypoxia
**Tollip**	4.20E-03	–1.21	Cytokine receptor binding, growth factor receptor binding
**Irf2**	5.56E-03	–1.26	Innate immunity, gene transcription, cell proliferation
**Lrp1**	5.63E-03	–1.32	Receptor-mediated endocytosis, regulation of cholesterol efflux, ECM
**Yy1**	6.44E-03	–1.21	Cellular response to DNA damage stimulus, cellular response to interleukin-1
**C3ar1**	6.95E-03	–1.47	Leukocyte chemotaxis, regulation of vasculature development
**Bcl6**	7.41E-03	–1.41	Regulation of immune response, Th2 cell differentiation
**Ifnar1**	9.03E-03	–1.25	Type I interferon receptor, innate immunity
**Cfb**	9.24E-03	–2.05	Activation of immune response, blood microparticle, inflammation
**Cd84**	9.32E-03	–1.79	Regulation of macrophage activation, positive regulation of MAPK cascade

Total tumor RNA was hybridized to mouse pancancer immune codeset (nanoString; *n* = 6 per treatment group).

## DISCUSSION

MMP-14 is highly expressed in metastatic cancers, is associated with poor prognosis, and a proven clinical target. Several groups have developed selective MMP-14 inhibitory antibodies that target either the catalytic domain or a collagen binding domain, and limit TNBC tumor growth and metastasis in xenograft assays [14, 26, 27]. To build on these efforts, we report here that several novel inhibitory synthetic antibodies were identified by phage display screening using the MMP-14 ECD. This includes a lead antibody (3369) that inhibits MMP-14 activity via binding within the catalytic domain. Fab 3369 shows low nM affinity for MMP-14 *in vitro*, and blocks MMP-14 activity towards ECM *in vitro* and *in vivo*. Treatment with this inhibitory antibody in the human IgG1 format was effective in limiting TNBC tumor growth and metastasis in mouse models. The mode of action for our MMP-14 inhibitory antibody is consistent with disruption of MMP-14 effects on tumor angiogenesis, hypoxia, immune suppression, and metastasis. Overall, our study builds on a recent study of mouse tumors engrafted in nude mice [27], and shows that MMP-14 blockade within breast tumors and TME limits tumor progression and metastasis.

Towards the generation of more potent binders based on our lead antibody (3369), we have completed screening of an affinity matured phage-displayed library derived from 3369, as described for our antibody development pipeline [28, 29]. Indeed, affinity maturation screening has recently yielded new anti-MMP-14 Fabs with significantly improved affinity (unpublished results). To improve the selection of epitopes exposed in activated MMP-14, we also plan to extend our screening to an activated MMP-14 Fc-ECD construct. Based our results obtained with Fab 3369, we expect that second generation antibodies will achieve a more complete blockade of MMP-14 in a number of metastatic cancer models.

A major advance for our study of MMP-14 blockade over the previous studies with DX-2400 inhibitory antibody [14, 27], is the testing of our inhibitory MMP-14 antibody in a syngeneic model of metastatic breast cancer. In fact, MMP-14 blockade in 4T1 tumors resulted in differential expression of immune regulatory genes and markers. The loss of Arg1 expression is consistent with MMP-14 blockade promoting an M2 to M1 shift in TAM phenotypes. The chemokine Ccl6 was highly downregulated as well, and this is notable because it is a target of the Myc oncogene and promotes tumor growth and metastasis [30]. Immune suppressive myeloid cells and granulocyte markers were also differentially expressed (Fcgr2b, encoding the inhibitory IgG receptor; Itgam, encoding Mac-1 integrin). It is also worth noting that Cathepsin S (encoded by Ctss) was downregulated, and has been implicated in generating angiogenic factors during tumor progression [31]. This may explain the reduction in CD31 tumor vasculature and tumor growth we observed with IgG 3369 treatment. The reduced levels of the hypoxia marker CA9 with MMP-14 blockade may relate to less vessel dysfunction as well. We expect that differential expression of some of these genes may be due to differences in density or activation of immune cells within the TME with MMP-14 blockade. Indeed, staining of immune cell markers in 4T1 tumor tissue sections revealed that MMP-14 blockade led to a skewing of the TME. This included more anti-tumor immune profiles, including increased density of iNOS+ cells and Granzyme B+ cells with MMP-14 blockade. Further studies using multiple markers will be needed to fully characterize the effects of MMP-14 inhibitory antibody treatments on immune subsets in the TME. Given the emergence of immune checkpoint inhibitor therapies in some cancers [32, 33], it will be important to test whether MMP-14 blockade may synergize with these therapies in the future. Indeed, combination therapies that include MMP-14 blockade may yield a more rigorous response to both immune checkpoint inhibitors and other standard therapies as well.

## MATERIALS AND METHODS

### MMP-14 extracellular domain expression, immunoblotting, and phage display screening

A mammalian expression construct was designed for expression of amino acids 23–534 of human MMP-14 extracellular domain in frame with IL-2 signal sequence at the N-terminus and human IgG1 Fc domain at the C-terminus using the vector pFuse2 (In*vivo*Gen, San Diego, CA, USA). The construct was expressed and affinity purified from conditioned media of HEK293F cells (In*vivo*Gen) following transient transfection. The ECD-Fc fusion protein was used as the antigen to screen a phage displayed synthetic humanized Fab library [21]. Binding selections, phage ELISAs and Fab protein purification were performed as described [28, 29]. Briefly, 10^13^ phage from a synthetic Fab library [21] were cycled through rounds of binding selection with the antigen immobilized on 96-well Maxisorp Immunoplates (Fisher Scientific, Nepean, ON, Canada) as the capture antigen. After four rounds of positive selection, preceded by negative selection using human IgG1 Fc domain alone, phages from round 4 were produced as individual Fab-phage clones in 96-well format and Fab-phage ELISAs were performed to detect specific binding clones. Clones with positive binding to MMP14-Fc compared to Fc domain were subjected to DNA sequencing. The genes encoding for variable heavy- and light-chain domains of unique positive clones were subcloned into vectors designed for production of Fab or IgG1 proteins in *Escherichia coli* or transfected HEK293F cells, respectively, and proteins were affinity-purified on Protein A columns (GE Healthcare, Mississauga, ON, Canada).

### Surface plasmon resonance (SPR)

SPR measurements were performed using a Bio-Rad ProteOn XPR instrument. MMP14 ECD-Fc fusion protein (180 RUs) was captured on a GLC sensor chip with immobilized anti-Fc antibody (Jackson ImmunoResearch cat # 109-005-098). Five three-fold serial dilutions of Fab protein in PBS, 0.05% Tween20 (starting at 200 nM) were injected for 120 seconds to monitor association, followed by injection of PBS, 0.05% Tween 20 for 900 seconds to monitor dissociation. Experiments were performed at 25°C. Sensorgrams were fit globally to a 1:1 binding Langmuir model using Bio-Rad ProteOn Manager fitting software.

### MMP-14 activity assays

The effects of anti-MMP-14 Fabs on MMP-14 protease activity was measured using a fluorimetric assay (MMP-14 Fluorescent Assay Kit for Drug Discovery AK-417, Biomol, PA, USA). In this assay, the purified catalytic domain of MMP-14 that was provided (lacking Hpx, prodomain and hinge region), was incubated with or without anti-MMP-14 Fabs (800 nM) prior to addition of a quenched fluorogenic substrate peptide (OmniMMP^TM^, Biomol) in black 96-well microtitre plates (Costar, Corning, NY, USA) using a fluorescence plate reader at 37°C. Fluorescence at 420 nm was measured (excitation wavelength of 328 nm) for 10 minutes at one-minute intervals. The change in initial rate was measured and expressed as a relative activity compared to control with gammaglobulin using the equation: RFU(Fab)-RFU(control)/RFU(control). In addition, we tested the effects of anti-MMP-14 Fab treatment on our larger MMP-14 Fc-ECD purified protein (same as bait protein for phage display screening) following activation by proteolyis of prodomain using trypsin (5 μg/ml for 60 minutes at room temperature), and using the same quenched fluorogenic substrate peptide.

### Cell lines and gene silencing

Human MDA-MB-231 and mouse 4T1 breast cancer cell lines were obtained from American Type Culture Collection. MDA-MB-231 cells with stable expression of constitutively active Src (MDA-Src cells) were described previously [22]. Briefly, Dulbecco's modified Eagle's medium (DMEM) supplemented with 10% fetal bovine serum (FBS) was used for MDA-MB-231 and MDA-Src cell cultures. RPMI 1640 supplemented with 10% FBS was used for 4T1 cell cultures. All cell cultures were maintained at 37°C in a humidified atmosphere of 5% CO_2_, 95% air. The stable silencing of MMP-14 was carried out using a pLKO.1 lentiviral construct encoding an shRNA targeting human MMP-14 from the TRC library (Dharmacon). Lentiviral production in transfected HEK293 cells, and the transduction and selection of MDA-MB-231 and MDA-Src were performed as previously described [34]. The extent of MMP-14 KD was evaluated by immunoblot with an MMP-14 antibody (Abcam).

### Cell growth assays

MDA-MB-231 and 4T1 cells were seeded in 96 well plates at low confluency, treated with either media alone, control IgG, Fab 3369, IgG 3369 (all at 500 nM in triplicate). The plate was placed in an IncuCyte ZOOM (Essen Biosciences) and images acquired every 2 hours for a period of 48 hours. The cell densities for each treatment group were calculated using imaging software.

### Flow cytometry analysis with Fab and IgG

MDA-MB-231, MDA-Src and 4T1 cells were grown to 80–90% confluence in the recommended media and harvested by trypsinization. Cells (10^6^) were washed twice in ice cold PAB buffer, incubated with Fc block (anti-CD16/32) for 5 min, followed by incubation with either Fab or IgG (5 ug/ml) for 30 min at 4°C. After two washes with 1 ml of PAB buffer, bound Fab or IgG was detected by the addition of 50 μl (1:100 dilution) of Alexa Fluor^®^ 488-AffiniPure F(ab′)_2_ Goat Anti-Human IgG (H+L) (Jackson ImmunoResearch). After incubating 30 minutes at 4°C, the cells were washed twice and resuspended in 500 uL of PAB buffer. Fluorescence was measured by flow cytometry using a FC 500 flourimeter (Beckman Coulter, Inc.), and median fluorescence intensity (MFI) was calculated using CXP software.

### Extracellular matrix degradation and cell invasion assays

MDA-MB-231 cells were treated with or without Fab 3369 or IgG 3369 at the indicated doses upon seeding on coverslips coated with TRITC-labeled thin gelatin for 18 hours, as previously described [34]. Degradation spots from epifluorescence micrographs were scored using imaging software (Image ProPlus). For the transwell invasion assay, the insert membranes (24-well inserts, 8.0 μm; Corning) were precoated with 50 uL of 20% growth factor-reduced matrigel (BD Biosciences). MDA-MB-231, MDA-Src or 4T1 cells were lifted from the plates with 10 mM EDTA in PBS. A total of 2.5 × 10^4^ cells in DMEM or RPMI media, respectively, were seeded into the top chambers of Transwell and treated with either IgG 3369, Fab 3369 or an irrelevant IgG at various concentrations. The bottom part of the chamber was filled with culture medium supplemented with 10% FBS. After 18–24 h of incubation at 37°C, the non-invading cells present on the upper surface of the insert were removed with a sterile cotton swab. The cells that invaded onto the lower surface of the filter were fixed with paraformaldehyde and stained with DAPI and filters were mounted onto glass slides. The number of cells that had invaded per membrane from five random fields were scored using an Olympus BX51 epifluorescence microscope equipped with a Q Color5 digital camera (100Å~/1.30 oil objective; images were acquired using QCapturePro software).

### Tumor models

Mammary orthotopic xenograft assays were performed using MDA-MB-231 cells implanted in Rag2^–/–^: IL2Rɣc^–/–^ (BALB/c) mice, as described previously [24, 34]. Briefly, mammary fat pads were injected with 1.5 × 10^6^ MDA-MB-231 cells in a 50% Matrigel (BD Biosciences) mixture. When mammary tumors were palpable, mice were randomized between intraperitoneal injections with either irrelevant negative control IgG (gammaglobulin) or IgG 3369 (5 mg/kg, *n* = 3–4 animals per group) every 2–3 days. Tumor volumes were calculated from measurements of tumors using digital calipers using the formula ½(length × width^2^). At endpoint (day 34), primary tumors were excised and weighed. Tumors and other tissues (lung, liver) were collected and either prepared as formalin-fixed, paraffin embedded tissues, or snap frozen and prepared for cryosectioning. For comparison to IgG 3369 treatments effects on xenograft tumor growth, parallel studies were performed with MDA-MB-231 vector and MMP-14 KD cells injected in mammary fat pads as described above. Syngeneic mammary orthotopic tumor studies were performed using 4T1 cells (10^3^) injected in mammary fat pads of Balb-c mice as described previously [25]. Upon the detection of palpable tumors, mice were treated with control IgG or IgG 3369 as described above, and tumor volumes analyzed until endpoint was reached. Tissue collection and analyses were similar to those described above with the exception of the collection of peripheral blood by cardiac puncture for a cohort of control IgG and IgG 3369 (*n* = 3/group). The blood samples were collected in EDTA-coated vacuum tubes and analyzed ANTECH Diagnostics (Mississauga, ON).

### Immunostaining

Frozen 20-μm sections were post-fixed in acetone and blocked for 1 hour with 3% bovine serum albumin prior to addition of primary antibodies overnight at 4°C. Sources and dilutions of primary antibodies were as follows: rabbit anti-CD31 (endothelial marker, 1:5,000; antisera described previously [35]), rabbit anti-Collagen IV (basement membrane marker; Millipore, AB7569, 1:1000), rabbit anti-Collagen I ¾ fragment (US Biologicals, catalog no. 207128), mouse anti-iNOS-FITC (BD Biosciences, 610330, 1:100), rabbit anti-CA9 (hypoxia marker; Abcam, ab15086, 1:3000), rat anti-CD206/MRC1-PE (M2 marker; Serotec, MCA2235, 1:50), rabbit anti-Granzyme B (cytotoxic activity marker; Abcam, ab4059, 1:200), and rabbit anti-Ki67 (proliferation marker; Abcam, ab15580). Detection of unconjugated antibodies was achieved using Alexa Fluor^488^-conjugated goat anti-rabbit secondary antibody staining (1:200) for 60 min at room temperature (DAPI was also included to detect cell nuclei, 1:400).

### NanoString nCounter gene expression analysis

NanoString gene expression profiling was performed on RNA extracted from MDA-MB-231 or 4T1 tumor xenografts (Trizol extractions) using the nCounter PanCancer Progression panel (human) or nCounter PanCancer Immune Profiling panel (mouse), respectively (NanoString Technologies, Seattle, WA, USA). The human PanCancer Progression panel contained specific probes to 740 endogenous genes and 30 housekeeping genes, and the mouse PanCancer Immune Profiling panel had probes specific to 750 endogenous genes and 20 housekeeping genes. Both panels also contained 6 positive controls with concentrations ranging between 0.125–128 fM and 8 synthetic negative control sequences. Briefly, 100 ng of total RNA isolated from primary tumors was hybridized to specific capture and barcoded reporter probes before being immobilized on a cartridge. A nCounter Digital Analyzer was used to count the fluorescent barcoded probes to quantify each target RNA molecule. The barcoded images captured by the automated fluorescent microscope were pre-processed for quality control metrics based on field of view (FOV) registration and binding density. All samples had FOV registration greater than 75% and binding densities within a 0.05–2.25 range, and therefore fell within standard quality specifications.

Processing and normalization of raw NanoString gene expression data was based on the steps outlined in the NanoStringNorm Bioconductor package [36]. First, normalization factors based on the 6 positive controls were calculated for each lane by dividing the geometric means of the positive controls across all lanes by the geometric mean of each given lane. The human MDA-MB-231 and mouse 4T1 tumors had positive control scale factors that fell between 0.81–1.27 or 0.57–1.6, respectively, well within the accepted range of 0.3–3. Next, a background subtraction was performed using the mean of the 8 negative controls across all lanes, plus two standard deviations. This more conservative subtraction was chosen to account for variability in background, and to filter out very low abundance genes that were likely not biologically relevant. Finally, sample content normalization factors were calculated in the same manner as for the positive controls, using the geometric mean of the housekeeping genes that showed the best reproducibility across the samples (18 genes for the human panel and 8 genes for the mouse panel). The positive control-normalized and background subtracted data was then multiplied against the sample content normalization factors to produce the final normalized gene expression data.

To identify genes that were significantly differentially expressed between the IgG 3369 and control treatment groups, the Welch *t*-test assuming unequal sample variances was employed. Cut-offs of *p ≤*0.01 and *p* ≤0.05 were selected to provide a list of high-confidence genes, and to identify all potential genes of interest. Genes determined to be significantly differentially expressed were entered into the KEGG (Kyoto Encyclopedia of Genes and Genomes) Mapper pathway mapping tool to identify pathways that were enriched. Differential expression data for genes in pathways that were found to be enriched was then overlaid onto KEGG pathway maps using the pathview Bioconductor package [37].

### Statistical analysis

Unless otherwise specified, all experiments were performed in triplicate and presented as the means ± standard error (SEM). The Student two-tailed t test was used to compare between treatment groups, with significant differences defined by *p* ≤0.05, unless otherwise stated in figure legends.

## SUPPLEMENTARY MATERIALS FIGURES AND TABLE


