# Supplementary material for: A novel immunotherapy targeting MMP-14 limits hypoxia, immune suppression and metastasis in triple-negative breast cancer models
Source: Oncotarget. 2017 May 9;8(35):58372–85. doi: 10.18632/oncotarget.17702 (PMC5601659; doi:10.18632/oncotarget.17702)
Supplement: Supplementary file 1 [file oncotarget-08-58372-s001.pdf]

A novel immunotherapy targeting MMP-14 limits hypoxia, immune suppression and metastasis in triple-negative breast cancer models

Supplementary Materials

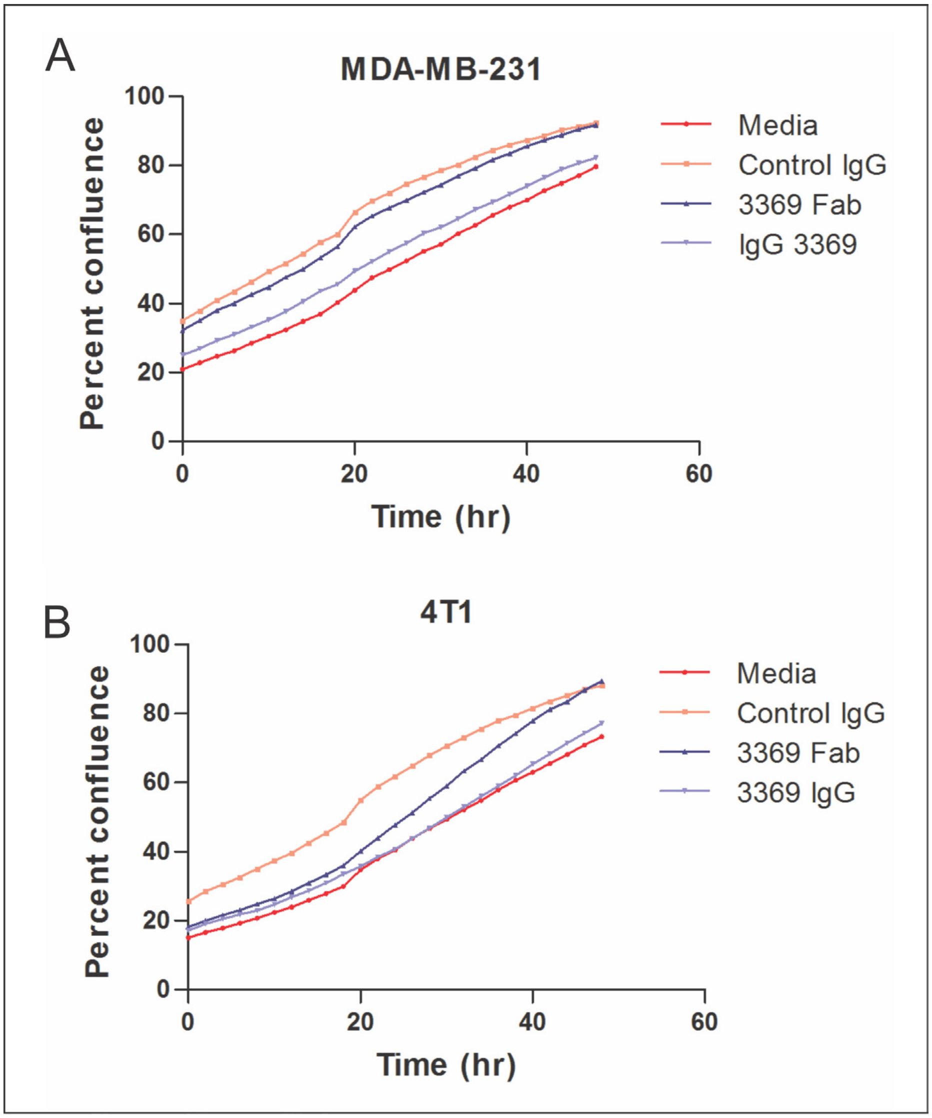

Supplementary Figure 1: Growth rates of mouse and human TNBC cell lines treated with Fab or IgG 3369. (**A–B**) MDA-MB-231 cells (A) or 4T1 cells (B) were seeded in 96 well plates at low density in triplicate. Cell densities were compared over 48 hours between untreated cells (Media), or those treated with control IgG, Fab 3369 or IgG 3369 (all at 500 nM) using an IncuCyte ZOOM (Essen Biosciences). Graphs depict the percent confluence for each treatment group.

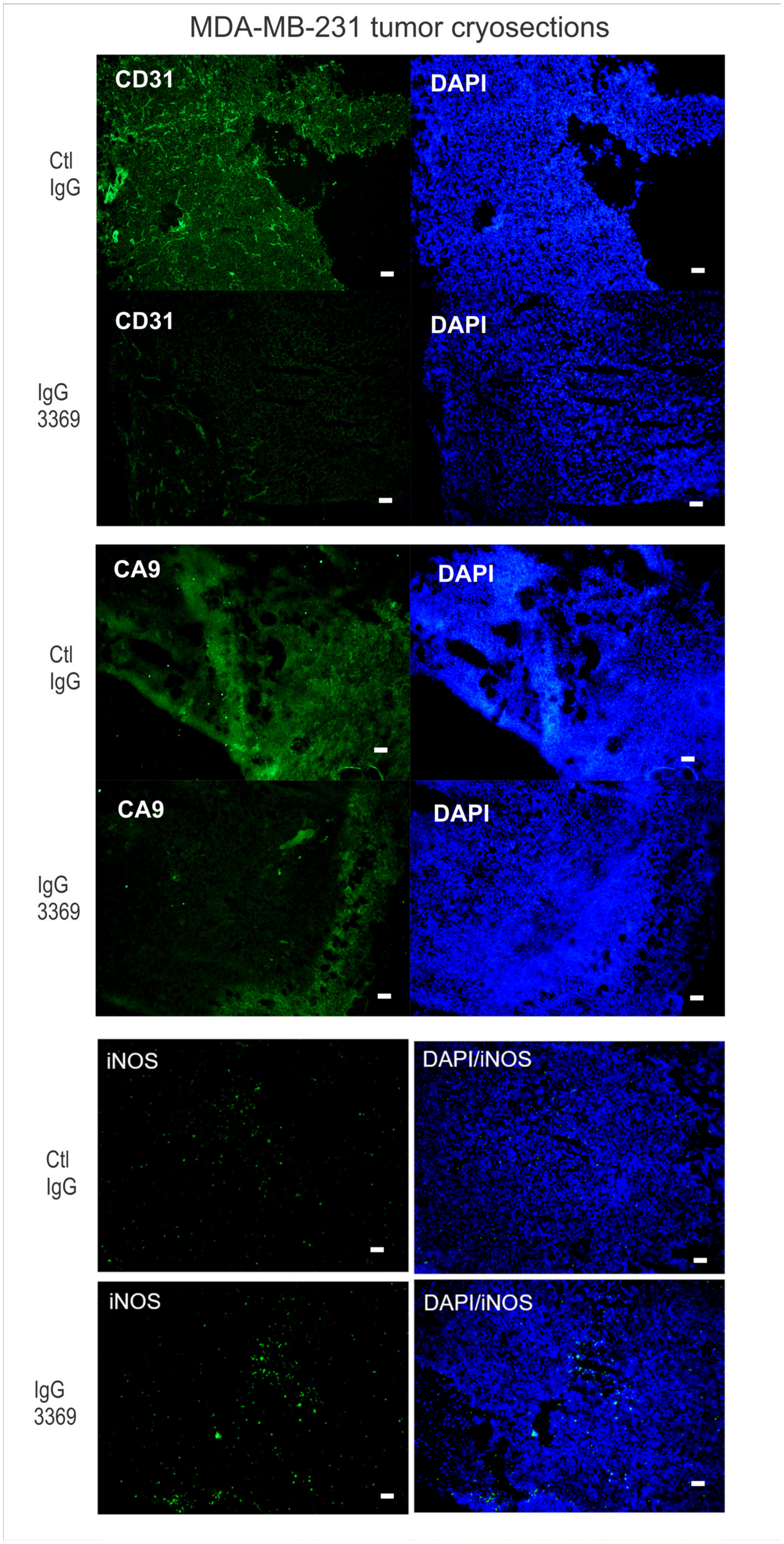

Supplementary Figure 2: Effects of anti-MMP-14 IgG 3369 treatment on tumor vasculature, hypoxia and macrophage markers. Representative images are shown for immunofluorescence staining of primary xenograft MDA-MB-231 tumor tissue cryosections from mice treated with control IgG or IgG 3369. The sources and dilutions of antibodies are described in Materials and Methods.

A

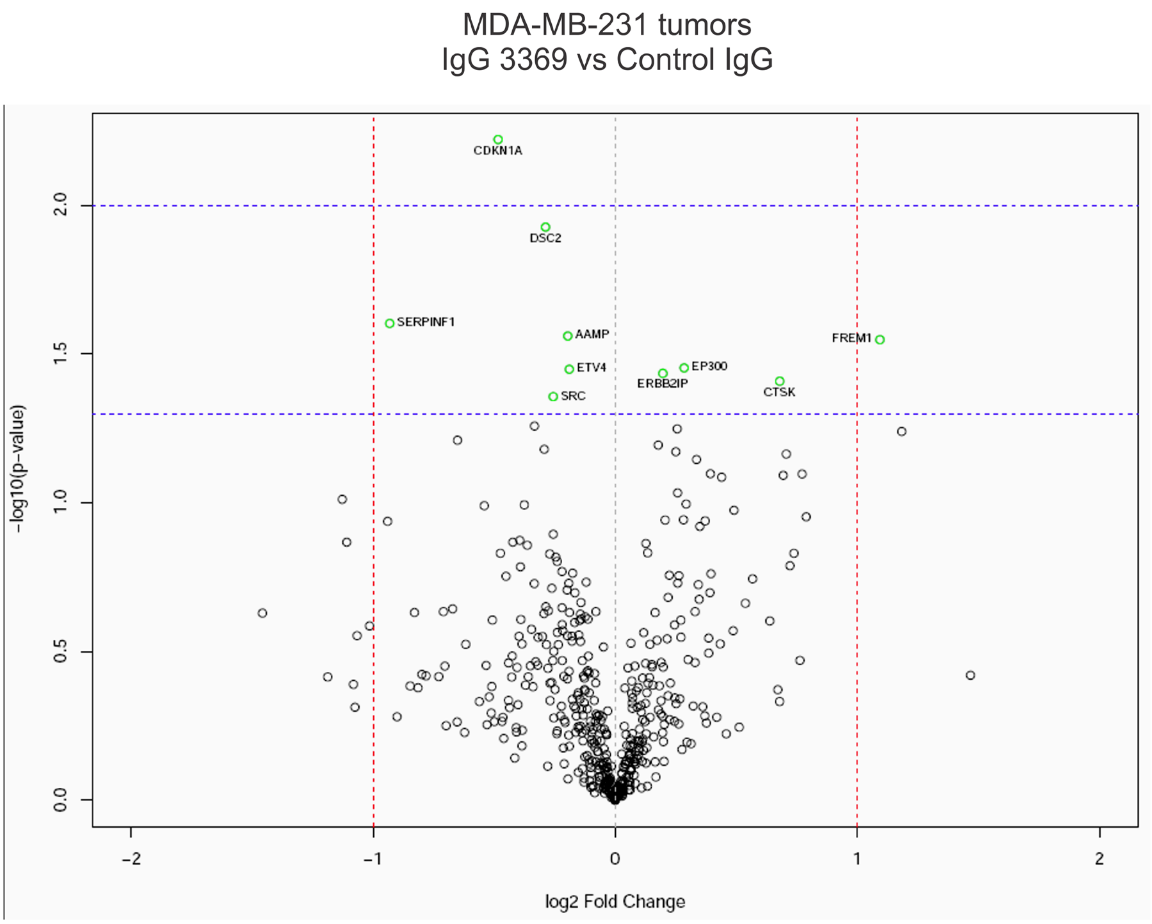

B

Gene	*p* value	Fold Change	Biological process
Cdkn1a	6.00E-03	-1.40	Cell cycle checkpoint, hypoxia, metastatic risk
Dsc2	1.18E-02	-1.22	ECM layers, EMT, TGFβ target, tumor invasion
Serpinf1	2.49E-02	-1.91	Angiogenesis, ECM layers, EMT, tumor growth
Aamp	2.75E-02	-1.15	Angiogenesis, ECM layers
Frem1	2.83E-02	2.13	ECM Layers, tumor invasion
Ep300	3.52E-02	1.22	Tumor growth, hypoxia, transcription factor
Etv4	3.56E-02	-1.14	Transcription factor, tumor growth, TGFβ target
Erbb2ip	3.67E-02	1.15	ECM layers, tumor growth, tumor invasion
Ctsk	3.90E-02	1.60	EMT
Src	4.40E-02	-1.19	Angiogenesis, ECM layers, tumor growth, tumor invasion

Supplementary Figure 3: MMP-14 blockade alters tumor progression and metastasis genes in TNBC tumors. (**A**) A volcano plot is shown to visualize differentially expressed genes in primary MDA-MB-231 tumors treated with IgG 3369 compared to treatment with control IgG based on nanoString profiling, as described in Materials and Methods (red dashed lines indicate > 2-fold change, and blue lines indicate $p < 0.05$ and $p < 0.01$ levels of significance). (**B**) Most significantly altered genes in MDA-MB-231 mammary tumors treated with anti-MMP-14 inhibitory antibody 3369 versus control IgG (Total tumor RNA was hybridized to human cancer progression codeset (nanoString; $n = 4$ per treatment group).

A

**KEGG Pathway**	**Genes**	***p*-value**
Phagosome	11	1.40E-06
Cytokine-cytokine receptor interaction	11	2.90E-05
Jak-STAT signaling pathway	7	1.20E-03
Complement and coagulation cascades	5	3.50E-03
Hematopoietic cell lineage	5	5.00E-03
PI3K-Akt signaling pathway	9	8.10E-03
Toll-like receptor signaling pathway	5	9.60E-03
Chemokine signaling pathway	6	2.30E-02
Antigen processing and presentation	4	3.00E-02
Cell adhesion molecules (CAMs)	5	4.50E-02
NF-kappa B signaling pathway	4	4.60E-02
Natural killer cell mediated cytotoxicity	4	5.60E-02

B

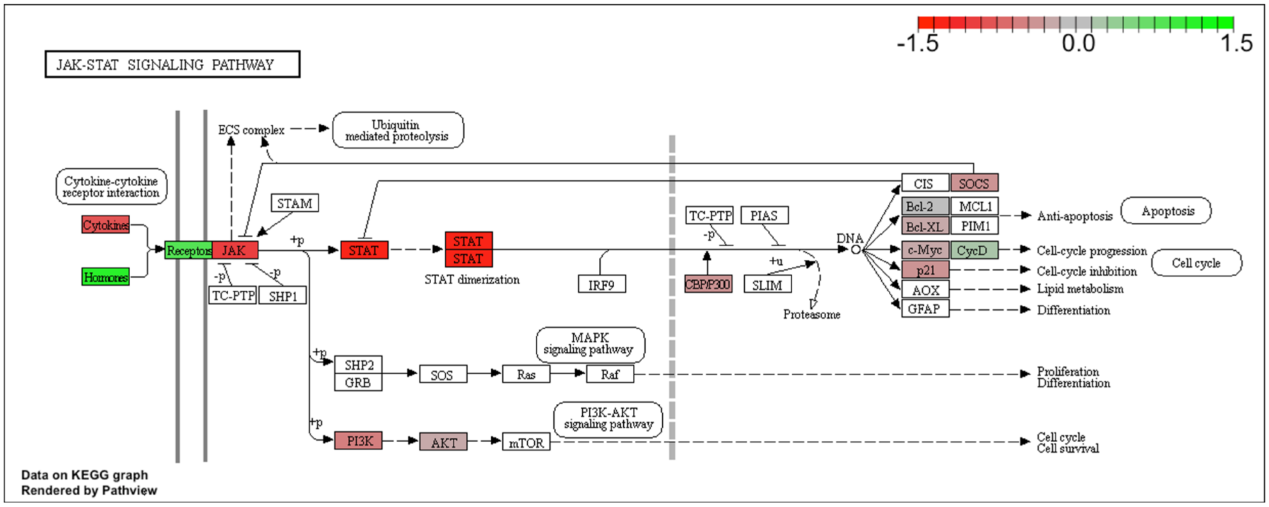

Supplementary Figure 4: MMP-14 blockade results in differentially expressed genes linked to immune and cytokine signaling pathways in 4T1 tumors. (**A**) Differentially expressed genes with anti-MMP-14 IgG 3369 treated versus control IgG treated 4T1 tumor-bearing mice were subjected to KEGG pathway analysis as described in Materials and Methods. Table includes a subset of the resulting pathways, along with number of genes affected in this pathway, and *p* values. (**B**) The fold changes in Jak-STAT pathway genes are shown as an example of MMP-14 treatment effects in the 4T1 tumor model.

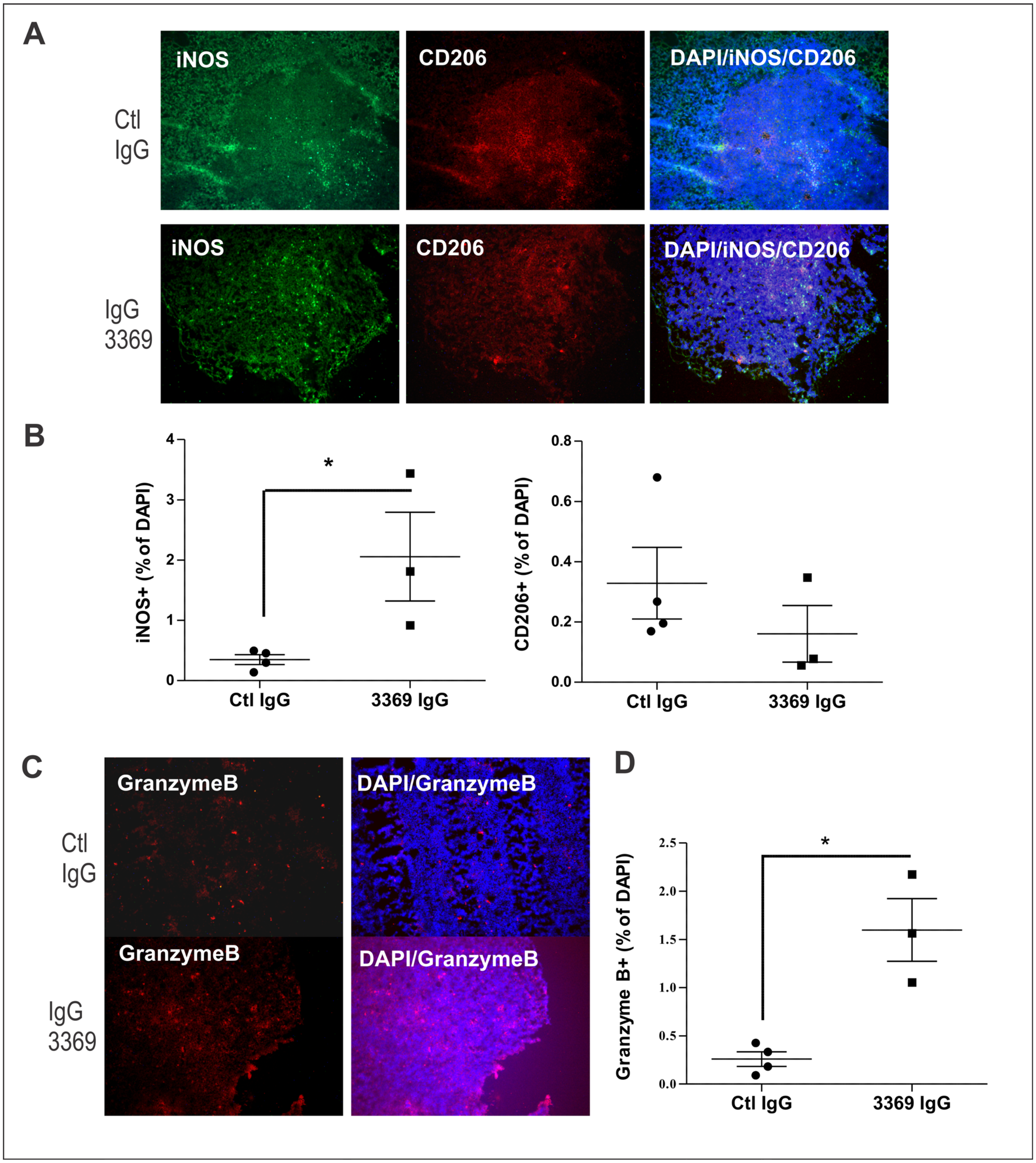

Supplementary Figure 5: MMP-14 blockade alters immune cell profiles in 4T1 tumors. (**A**) Immune cell subsets were analyzed in 4T1 tumors from mice treated with either control (Ctl) IgG and IgG 3369 by staining cryosections with anti-iNOS (green) and anti-CD206 (red) relative to DAPI-stained tumor area. Representative epifluorescence micrographs are shown. (**B**) Graph depicts the mean levels of iNOS+ and CD206+ cells for each treatment group (n = 3–4 animals per group, *indicates $p < 0.05$). (**C**) Staining of Granzyme B+ cells relative to DAPI-stained tumor area was performed as described above and representative epiflourescence micrographs are shown. (**D**) Graphs depict the mean levels of Granzyme B+ cells for each treatment group (n = 3–4 animals per group, *indicates $p < 0.05$).

Supplementary Table 1: No overt signs of toxicity with MMP-14 blockade in mice harboring 4T1 tumors

Parameter*	Control IgG	IgG 3369	*P* value
WBC (10^9/L)	36.6 ± 18.7	22.8 ± 8.8	0.31
RBC (10^{12}/L)	7.9 ± 0.7	7.3 ± 1.2	0.52
Hemoglobin (g/L)	125.7 ± 9.2	121 ± 14	0.66
Hematocrit (%)	42 ± 3.5	41 ± 5.3	0.79
MCV (fL)	53.3 ± 1.2	56.0 ± 2.7	0.18
MCH (pg)	16 ± 0	16.7 ± 0.6	0.12
MCHC (g/L)	299.7 ± 2.9	295.7 ± 8.7	0.49
Platelets (10^9/L)	1205 ± 116	1367 ± 151	0.21

*Peripheral blood was collected at endpoint from 4T1 tumor-bearing mice treated with control IgG (n = 3) or IgG 3369 (n = 3) and blood counts and hematological parameters provided (mean ± SD; *P* values based on a two tailed *T* test).
